# Synergy in IR–Hybrid CT/C-arm in the setting of critical trauma

**DOI:** 10.1007/s10140-022-02015-7

**Published:** 2022-02-02

**Authors:** Joseph A. Knox, Vishal Kumar, Miles B. Conrad, Sujal Nanavati, Teri Moore, Mark Wilson

**Affiliations:** 1 San Francisco Department of Radiology and Biomedical Imaging, University of California, San Francisco, CA 94110 USA; 2 San Francisco General Hospital, San Francisco, CA 94001 USA

**Keywords:** Hybrid computed tomography (CT), Trauma, Patient transport

## Abstract

Access to multi-detector computed tomography (MDCT) scanning for interventional procedures can prove to be logistically challenging as resources are often in different areas within the hospital. At some institutions, interventional radiology suites have moved to the operating room, separate from the diagnostic radiology department. At these institutions, complex interventional procedures requiring both fluoroscopy and MDCT may pose logistical challenges, especially as they pertain to timely patient transfers. Hybrid CT/fluoroscopy suite provides rapid, reliable MDCT assessment of trauma patients before and after emergent surgery, as well as access to the entire spectrum of emergent image-guided interventions in the same suite.

## Introduction

With the development of angiographic imaging and interventional techniques, interventional radiology (IR) is expected to provide an expanded role in the management of acute trauma patients [1]. Despite the increasing utilization of medical imaging technologies in the trauma setting, including multi-detector computed tomography (MDCT) and interventional radiology (IR), access to MDCT scanners for interventional procedures may prove logistically challenging as resources are often in different areas within the department, or in some cases, on different levels within the hospital [2]. At some institutions, IR angiography suites have moved to the operating room, separated from the diagnostic radiology department. For patients and physicians at these institutions, complex interventional procedures requiring both fluoroscopy and MDCT may pose logistical challenges. While negative outcomes pertaining directly to transport for IR procedures have not been studied, intra-hospital transport has been shown to be associated with negative outcomes in critically ill patients [3, 4].

In the setting of acute trauma, the benefits of whole-body diagnostic CT imaging on patient mortality are well established [5, 6]. With regard to vascular injury, MDCT has emerged as the standard for evaluation of patients with suspected vascular injury from blunt trauma [7, 8]. While some operating rooms (ORs) and angiography suites may come equipped with flat panel C-arm CT capabilities, in-room MDCT scanners allow for acquisition of multi-phase, diagnostic level imaging of nearly the entire patient’s body, without image cut-off, a major limitation of cone beam CT [9, 10]. The hybrid CT/C-arm system combines a flat-panel detector with a sliding gantry system, thereby allowing for safe and time efficient transitions between modalities without having to physically transfer the patient [11].

Serving as a safety net hospital, our institution is our city’s largest primary care facility, as well as a level 1 trauma center, treating more than 3900 trauma patients annually. In our hospital, MDCT scanners are available in the emergency department, which is located on the first floor (entry) level. Interventional radiology is located in the operating room on the ground level, one level below the emergency department and separate from diagnostic radiology. Diagnostic radiology is housed on the basement level, two floors beneath the emergency department, and one floor beneath the interventional radiology and operating room suites.

## Emergent intervention applications

**Fig. 1:**
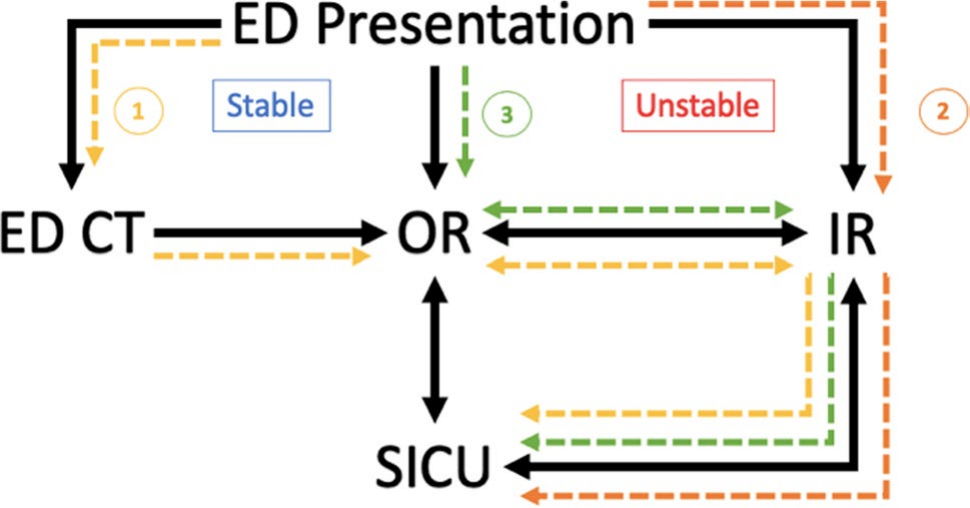
Trauma workflow at our institution, with three illustration cases (1, 2, 3). ED CT = Emergency Department CT scanner, ED Presentation = patient presenting to Emergency department, all on first floor of hospital (level 1). OR = operating room, IR = Interventional Radiology, both located one floor below ED, behind sterile barrier (ground floor). SICU = Surgical Intensive Care Unit, located above the first floor. Case presentations are indicated by numerical values

The hybrid CT/fluoroscopy unit allows for acquisition of cone beam CT as well as traditional MDCT for biopsies, aspirations and drainages. This inherent flexibility of the hybrid CT/fluoroscopy unit has allowed our IR service to undertake traditionally complex, multi-modality procedures in the operating room setting, while providing our trauma and other surgical colleagues with access to rapid MDCT assessment of trauma patients before and after emergent surgery. An illustration depicting the role of IR in the workflow of trauma patients at our institution can be seen in Fig. 1, with three example cases. Institutional Review Board approval was obtained for all cases.

## Case 1

A 50-year-old female presented with head trauma and hemodynamic instability after being struck by a motor vehicle. Initial CT scanning in the ED demonstrated severe pelvic fractures resulting in multiple foci of active extravasation, as well as subdural hematoma, which required emergent laparotomy and emergent hemicraniectomy. Following pelvic packing in the operating room suite, IR was consulted for pelvic angiography and embolization.

**Fig. 2:**
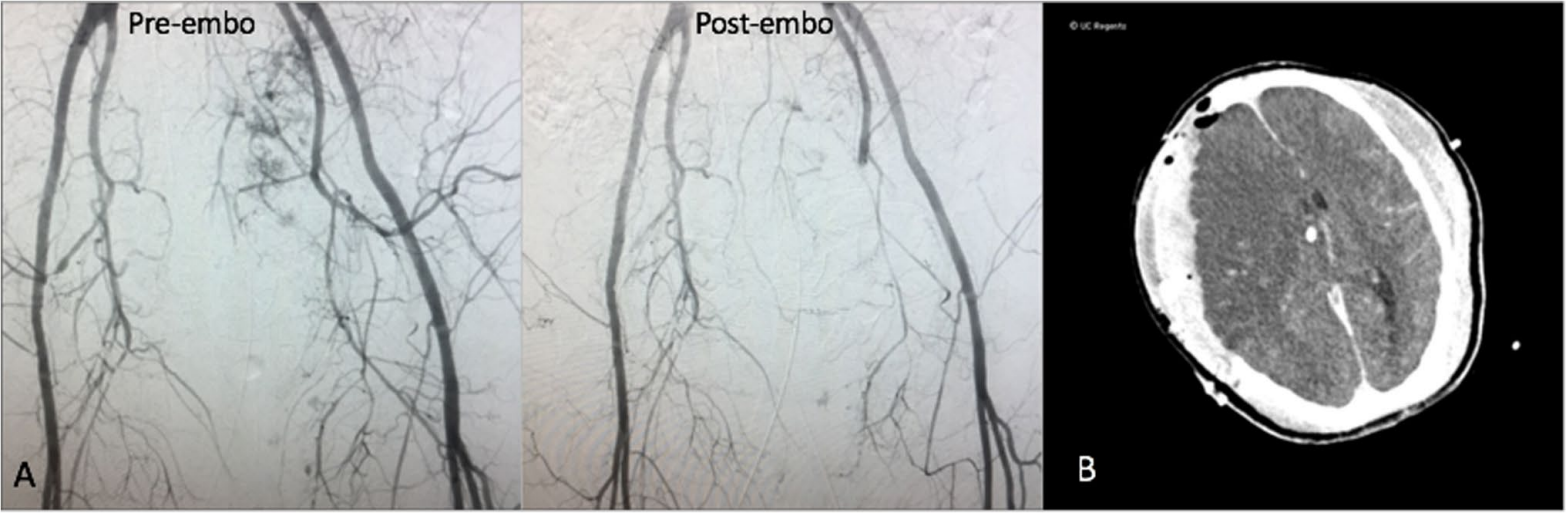
Pre and post embolization pelvic angiography (**A**) and head CT (**B**) of case 1

Acknowledging nursing concerns over repeated transports and transfers in such a critically ill patient, a follow-up diagnostic non-contrast head CT imaging (Fig. 2B) on the multi-detector system was obtained after emergent left transradial angiography and pelvic embolization (Fig. 2A). The workflow for this case is summarized by the yellow path labeled “1” in Fig. 1.

## Case 2

A 30-year-old male presented with complex pelvic fractures and hemodynamic instability following a pedestrian versus automobile accident. Given high suspicion for pelvic bleeding, the patient was transferred directly to the IR suite from the emergency department, with no cross-sectional imaging obtained.

**Fig. 3:**
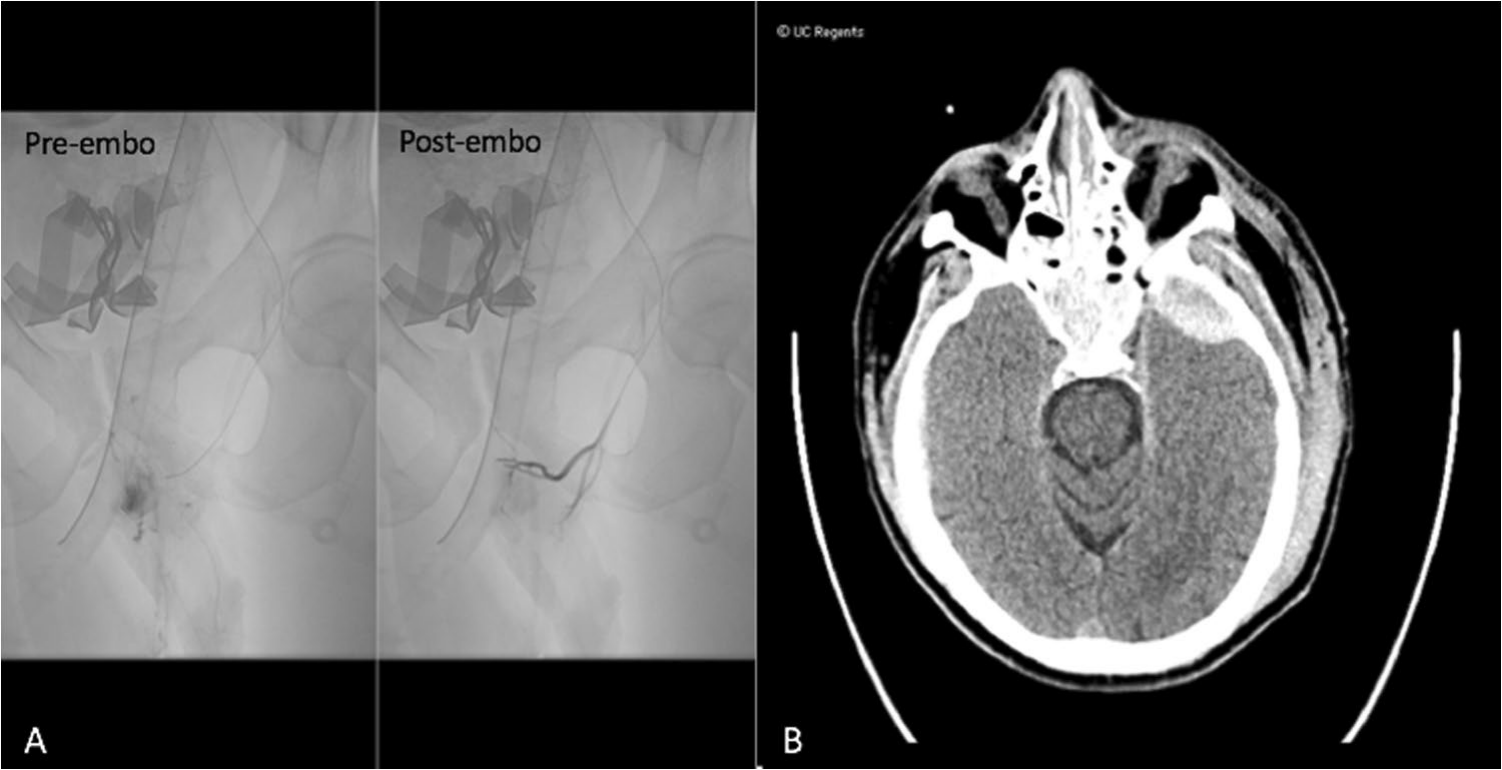
Pre and post pelvic angiography (**A**) and non-contrast head CT (**B**) of case 2

In IR, emergent transarterial pelvic angiography was performed, demonstrating active extravasation from branches of the left internal pudendal artery (Fig. 3A), which was successfully embolized with gelfoam slurry. Diagnostic arteriography also demonstrated trauma-related occlusion of the left tibial-peroneal trunk. At this juncture, the patient received a CT angiogram of the head, neck, chest, abdomen and pelvis, which revealed an epidural hematoma, left scapular fracture, and complex fractures of the pubic symphysis and left sacrum (Fig. 3B). As no additional acute life-threatening injuries were identified, successful endovascular recanalization of the occluded vessel was performed in the angiography suite. Post stent angiogram demonstrated successful thrombectomy and recanalization of the tibial peroneal occlusion, with restoration of posterior tibial and peroneal flow to the foot. The workflow of this case is summarized in the orange path labeled “2” in Fig. 1.

## Case 3

A 20-year-old male was brought in with multiple gunshot wounds to the right head, bilateral posterior chest, and right arm. On presentation, he was unresponsive with a GCS of 3. CPR was rapidly initiated, and given suspicion for massive bleeding, he was taken directly to the OR.

**Fig. 4:**
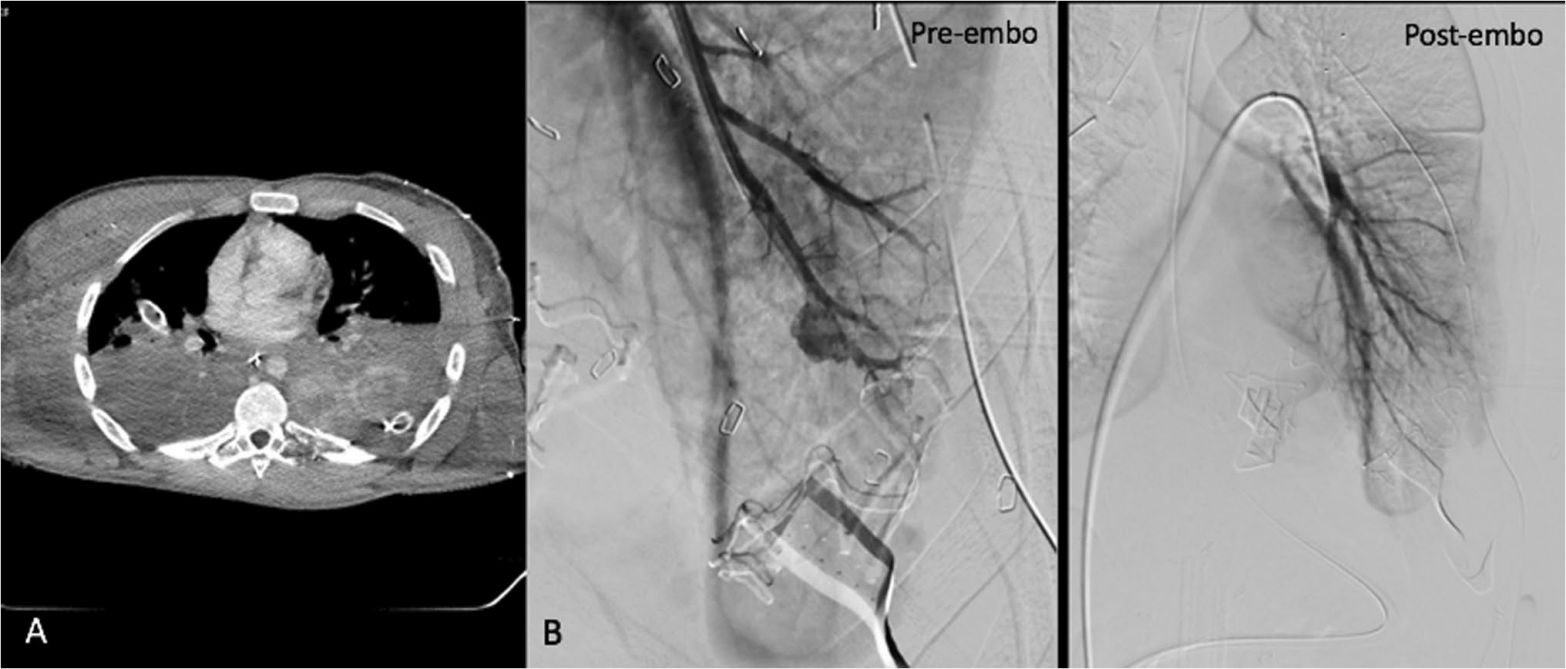
CT chest (**A**) and pre and post pulmonary angiography (**B**) of case 3

In the OR, bilateral chest tubes were placed, and left thoracotomy with packing was performed. Persistent hemoptysis compromised the patient’s airway, and as the source of bleeding could not be identified during thoracotomy, the patient was transferred to IR, where a CT angiogram of the chest, abdomen and pelvis, including delayed phase imaging, was obtained (Fig. 4A). Diagnostic imaging demonstrated massive pulmonary hemorrhage due to injury to the pulmonary artery, and endovascular embolization was performed in the IR suite (Fig. 4B). Following embolization, the patient was then taken back to the OR for exploratory laparotomy and splenectomy. The workflow for this case is summarized by the green path labeled “3” in Fig. 1.

## Discussion

The hybrid CT/fluoroscopy unit has allowed our service to undertake traditionally complex, multi-modality procedures in a safe and efficient manner. Furthermore, the hybrid system has provided our trauma and other surgical colleagues with access to rapid MDCT assessment of trauma patients before and after emergent surgery, as well as the full range of interventional procedures.

From an operators’ perspective, the hybrid unit allows for seamless transitioning between diagnostic CT imaging and image-guided intervention on highly unstable patients, a distinct advantage the room provides over traditional angiography suites. In cases 1 and 2 (Fig. 1), whole body diagnostic multi-detector CT imaging was obtained without leaving the IR suite, or the angiography table, obviating need for patient transfer and transport to the ED CT scanner, located on another floor of the hospital. Although necessary interventions on critically ill patients are not withheld due to risks of transport, intrahospital transport has been shown to be associated with higher morbidity and mortality [3, 12]. In addition to providing our surgical colleagues with rapid access to MDCT without necessitating transport across multiple floors, our interventionalists continue to integrate themselves into the multi-disciplinary approach required for the care of trauma patients. Finally, hybrid systems provide improved real-time imaging in the body compared to cone beam CT guidance, which may be especially important in challenging cases involving bowel motion artifact or bleeding [13]. The presented cases demonstrate the variety of ways in which a hybrid MDCT/fluoroscopy system has allowed for the integration of IR into the care of trauma patients at our institution.

There are limitations and disadvantages of the integrated hybrid system. Most significant is the increased patient radiation dose when using full diagnostic multi-detector CT as compared to cone beam CT. The diagnostic and clinical advantages of multi-phase imaging, and evaluation of multiple organ systems with one scan protocol, however, outweigh the theoretical risk of increased radiation dose. Another limitation is the lack of in-house IR technologists familiar with operating the system after-hours and on-weekends.

In conclusion, integration of a hybrid angiography-CT suite may provide benefit to the multi-disciplinary trauma care team, allowing IR to provide safe and efficient transitions between diagnostic imaging and interventional modalities.
